# Incipient dementia and avoidable hospital admission in persons with osteoarthritis

**DOI:** 10.1016/j.ocarto.2023.100341

**Published:** 2023-01-20

**Authors:** Ali Kiadaliri, L Stefan Lohmander, Leif E. Dahlberg, Martin Englund

**Affiliations:** a Clinical Epidemiology Unit, Orthopaedics, Department of Clinical Sciences Lund, Lund University, Lund, Sweden; b Centre for Economic Demography, Lund University, Lund, Sweden; c Department of Clinical Sciences Lund, Orthopaedics, Lund University, Lund, Sweden

**Keywords:** Avoidable hospitalization, Ambulatory-care sensitive conditions, Osteoarthritis, Incipient dementia, Register-based study, Sweden

## Abstract

**Objective:**

To investigate the associations between incipient dementia (ID) and hospitalization for ambulatory care sensitive conditions (ACSCs) among people with osteoarthritis (OA) of the peripheral joints.

**Methods:**

Among individuals aged 51–99 years residing in Skåne, Sweden, in 2009, we identified those with a doctor-diagnosed OA and no dementia during 1998–2009 (n ​= ​57,733). Treating ID as a time-varying exposure, we followed people from January 1, 2010 or their 60th birthday (whichever occurred last) until hospitalization for ACSCs, death, 100th birthday, relocation outside Skåne, or December 31, 2019 (whichever occurred first). Using age as time scale, we applied flexible parametric survival models, adjusted for confounders, to assess the associations between ID and hospitalization for ACSCs.

**Results:**

There were 58 and 33 hospitalizations for ACSCs per 1000 person-years among OA people with and without ID, respectively. The association between ID and hospitalization for any ACSCs was age-dependent with higher risk in ages<86 years and lower risks in older ages. Between ages 60 and 100 years, persons with ID had, on average, 5.8 (95% CI 0.9, 10.7), 1.6 (−2.6, 5.9) and 3.1 (2.3, 4.0) fewer hospital-free years for any, chronic and acute ACSCs, respectively, compared with persons without ID.

**Conclusions:**

Among persons with OA, while ID was associated with increased risks of hospitalization for ACSCs in younger ages, it was associated with decreased risk in oldest ages. These results suggest the need for improvement in quality of ambulatory care including the continuity of care for people with OA having dementia.

## 1 Introduction

Osteoarthritis (OA) is a leading cause of pain and disability which in turn translates into increased healthcare use and productivity losses [[1], [2], [3]]. The coexistence of chronic conditions is very common in OA with about two in three persons with OA having one or more co-existing conditions [[4], [5], [6]]. One such co-existing condition is mental illness including anxiety and depression [7]. A recent meta-analysis suggested that OA might also be associated with an increased risk of dementia [8]. Co-existing conditions increase the complexity of disease management and can lead to poor quality of care including hospital admissions for ambulatory care sensitive conditions (ACSCs) [9,10], an established measure of primary care quality [11]. ACSCs include several chronic and acute conditions that can be effectively managed in outpatient setting [12]. The assumption underlying the ACSCs concept is that appropriate and timely preventive care and disease management, generally provided in ambulatory setting, should avoid the need for costly hospital admissions [13].

Given that ACSCs can be potentially avoided through the provision of high quality ambulatory care, monitoring ACSCs among patient subpopulations can provide useful information for targeted interventions toward those most in need of improvement in outpatient care [14]. A recent population-based study reported that OA is associated with an 11% increased hazard of hospital admission for ACSCs [15]. Previous studies also suggested that mental illness including dementia is associated with elevated risk of hospital admission for ACSCs [[16], [17], [18], [19], [20]], although the evidence is mixed [9,21,22]. There is, however, limited evidence on implication of incipient dementia for healthcare use and quality of care among individuals with OA. This study aimed to fill this knowledge gap by investigating the association between comorbid dementia and *quality of care*, measured by ACSCs, in persons with OA using high-quality longitudinal population-based register data from the southern Sweden. Considering health and economic burden of poor quality of care, identifying individuals at high risk of poor quality of care have significant implications for patients’ health and healthcare resource optimization.

## 2 Methods

### 2.1 Study design and data sources

We conducted an observational population register-based cohort study in Skåne, the southernmost region of Sweden. We obtained the data on age, sex, date of death and residential addresses from the Swedish Population Register. We retrieved individual-level data on highest educational attainment, income, country of birth, marital status and parents’ immigration status from the Longitudinal Integration Database for Health Insurance and Labour Market Studies (LISA by Swedish acronym). Data on all health-care consultations (public and private) in the region were extracted from the Skåne Healthcare Register (SHR), a regional legislative administrative healthcare database. SHR contains the diagnostic codes from publicly funded clinics according to the International Classification of Diseases 10 (ICD-10) system assigned by physicians at the time of the healthcare consultation. We linked all these registers using the unique personal identification number, which was replaced with an arbitrary code by the Swedish authorities to ensure the anonymity of the subjects. The study was approved by the Regional Ethical Review Board in Lund, Sweden (Dnr 2011–432 and 2014–276).

### 2.2 Study cohort

We identified all individuals aged 51–99 years resided in Skåne on December 31, 2009 who had been living there since January 1, 1999 (n ​= ​404,002). To minimize potential confounding due to propensity to seek care, we excluded 3530 individuals with no in-person healthcare consultation recorded in the SHR during 1998–2009. Among remaining, we identified persons with a doctor diagnosed OA at peripheral joints (ICD-10 codes M15–M19) as the main diagnosis from the SHR during 1998–2009 (n ​= ​59,434). We then excluded 1701 individuals with a dementia diagnosis (ICD-10 codes F00–F03, F051, G30, G311) as main or secondary diagnoses during 1998–2009, resulting a final sample of 57,733 individuals with OA and no dementia at the baseline.

### 2.3 Exposure

Incipient dementia defined as a new diagnosis of dementia as the *main diagnosis* in the SHR between January 1, 2010 and December 31, 2019 was used as *the exposure of interest*. Incipient dementia was treated as a time-varying exposure meaning that people with dementia were treated as unexposed during the period prior to diagnosis.

### 2.4 Outcome and follow up

The outcome was a hospital admission for ACSCs in the SHR. To identify such hospital admissions, we relied on the definition of avoidable hospitalization developed by the Swedish National Board of Health and Welfare and Swedish Association of Local Authorities and Regions [23] (Table A1 in supplement). Accordingly, we considered anemia, angina, asthma, chronic obstructive pulmonary disease (COPD), diabetes, heart failure, and hypertension as *chronic ACSCs* and bleeding gastric ulcer, diarrhea, ear, nose and throat infection, epileptic seizures, inflammatory diseases of female pelvic organs, and pyelonephritis as *acute ACSCs*. We studied any ACSCs, chronic ACSCs, acute ACSCs, and 6 more common specific ACSCs: angina, COPD, diabetes, heart failure, epileptic seizures, and pyelonephritis. Each participant was followed from January 1, 2010 or 60th birthday (whichever occurred last) until hospital admission for the outcome of interest, death, 100th birthday, relocation outside Skåne, or December 31, 2019, whichever came first. We started follow up from the 60th birthday since dementia is less common in younger ages.

### 2.5 Data analysis

We used the parametric survival models based on restricted cubic splines with 5° of freedom to model the baseline hazard and to estimate hazard ratio (HRs) with 95% confidence intervals (CIs) (using Stata's “stpm2” command) [24,25]. Attained age was used as the time scale. To account for (potential) non-proportional hazard, we estimated models with time-dependent coefficient for incipient dementia with up to 3° of freedom and compared these with time-constant coefficient (proportional hazard) and selected the final model based on the Bayesian information criterion (BIC) and model parsimony. Using final models, we also predicted two standardized survival curves under two counterfactuals: one in which no one in our sample was exposed to incipient dementia and one in which everyone was exposed to incipient dementia (using Stata's “stpm2_standsurv” command). We then calculated the difference between the areas under these survival curves which reflect the difference in hospital-free years associated with incipient dementia between ages 60 years and 100 years.

**Fig. 1:**
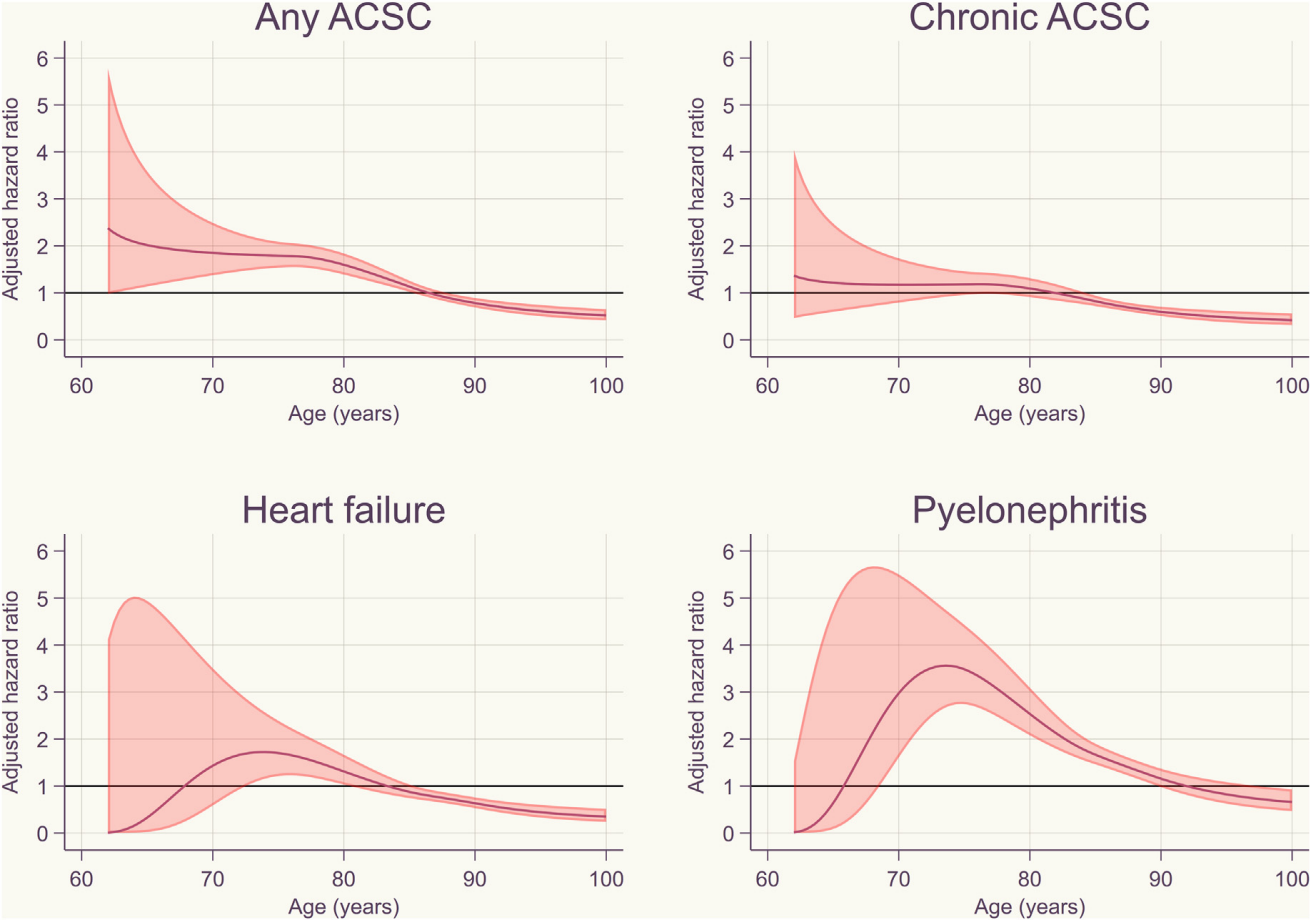
Age-dependent adjusted hazard ratios for the associations between incipient dementia and hospital admission for ambulatory-care sensitive conditions.

All models were also adjusted for sex, age (50–64, 65–74, 75–84, 85+), marital status, educational attainment, income, prior hospital admission for ACSCs during 1998–2009, Charlson comorbidity index during 1998–2009, and participant's as well as her/his parents' country of birth (born in Sweden vs. abroad); all measured at baseline. Subgroup analyses by sex and among those with no previous hospital admission for ACSCs during 1998–2009 were conducted.

## 3 Results

After excluding two persons with missing country of birth, a total of 57,731 people with mean (SD) age 70.6 (10.5) years, 61.1% females, were included. Of these, 4630 (8.0%) were diagnosed with a dementia during 2010–2019. Older people, females, persons with lower education and income and those with more comorbidities at baseline were more likely to have a dementia diagnosis during the follow up (Table 1).

**Table 1:** Baseline characteristics of the participants (December 31, 2009).

N	No dementia	Dementia over follow up
	53,101	4630
Age at entry (years), mean (SD)	69.9 (10.5)	78.2 (7.4)
Females, %	60.6	67.2
Level of education, %		
0–9 years of education	39.2	49.3
10–12 years of education	40.4	35.0
≥13 years of education	19.5	14.3
Missing	0.9	1.4
Marital status at entry, %		
Never married	7.6	5.0
Previously married	36.3	48.7
Married	56.1	46.3
Born in Sweden, %	88.1	86.9
Mother born in Sweden, %	86.4	86.5
Father born in Sweden, %	87.0	86.6
Income quartile, %		
Lowest quartile	24.7	29.8
Quartile 2	24.5	31.1
Quartile 3	25.0	24.7
Highest quartile	25.8	14.4
Charlson comorbidity index^a^, %		
0	50.8	40.1
1	20.7	23.8
2	13.0	17.1
≥3	15.5	19.0
Hospital admissions for ACSCs^a^, %		
0	84.9	81.1
1	6.9	8.4
2	3.2	4.7
≥3	5.0	5.8

ACSCs: ambulatory care sensitive conditions.

a Based on data from January 1, 1998 to December 31, 2009.

Crude rates of hospital admission for any ACSC were 33 and 58 per 1000 person-years among OA people without and with incipient dementia, respectively (Table 2). Among those without dementia, the rate for chronic ACSCs was greater than acute ACSCs (23 vs. 13 per 1000 person-years). On contrary, the rate for acute ACSCs was higher than chronic ACSCs among those with dementia (35 vs. 28 per 1000 person-years). Among persons without dementia, the greatest crude rate for a specific ACSC was observed for heart failure. On the other hand, pyelonephritis had the greatest crude rate among those with dementia.

**Table 2:** Number, rates, hazard ratios and hospital free years for ambulatory-care sensitive conditions in osteoarthritis patients with compared with those without incipient dementia.

	Number of events		Crude rate per 1000 person-years (95% CI)		Adjusted hazard ratio (95% CI)^a^	Hospital free years (95% CI)^b^
	No dementia	Dementia	No dementia	Dementia		
Any ACSCs	12,790	529	32.9 (32.3, 33.5)	57.8 (53.1, 63.0)	Fig. 1	−5.8 (−10.7, −0.9)
Chronic ACSCs	9140	296	22.8 (22.2, 23.3)	28.2 (25.1, 31.5)	Fig. 1	−1.6 (−5.9, 2.6)
Angina	2165	34	5.1 (4.9, 5.4)	2.9 (2.1, 4.0)	0.50 (0.36, 0.71)	2.1 (1.3, 2.9)
COPD	1440	43	3.4 (3.2, 3.5)	3.6 (2.7, 4.9)	0.77 (0.57, 1.05)	0.6 (−0.0, 1.2)
Diabetes	2182	76	5.1 (4.9, 5.4)	6.5 (5.2, 8.1)	1.08 (0.86, 1.36)	−0.2 (−1.0, 0.5)
Heart failure	4233	189	10.0 (9.7, 10.3)	16.4 (14.2, 18.9)	Fig. 1	0.2 (−1.2, 1.5)
Acute ACSCs	5379	366	12.9 (12.6, 13.3)	35.1 (31.7, 38.9)	1.58 (1.42, 1.76)	−3.1 (−4.0, −2.3)
Epileptic seizures	603	50	1.4 (1.3, 1.5)	4.2 (3.2, 5.6)	2.43 (1.81, 3.28)	−1.5 (−2.2, −0.8)
Pyelonephritis	3726	279	8.8 (8.6, 9.1)	25.4 (22.6, 28.6)	Fig. 1	−3.6 (−5.3, −1.9)

ACSCs: ambulatory care sensitive conditions.

a Adjusted for sex, age, marital status, educational attainment, income, prior hospital admission for ACSCs during 1998–2009, Charlson comorbidity index during 1998–2009, and participant's as well as her/his parents' country of birth. Time-varying hazard ratios are represented in Fig. 1 since a single HR cannot be reported in such situations.

b Adjusted for sex, age, marital status, educational attainment, income, prior hospital admission for ACSCs during 1998–2009, Charlson comorbidity index during 1998–2009, and participant's as well as her/his parents' country of birth. The difference between persons with and without dementia.

Our survival analysis suggested that the association between incipient dementia and hospital admission for *any* ACSC was age-dependent with higher risks in ages younger than 86 years and lower risks in older ages (Table 2, Fig. 1). In the absolute terms, incipient dementia was associated with 5.8 (95% CI 0.9, 10.7) fewer hospital-free years between ages 60 and 100 years. For *chronic* ACSCs, there was no association in ages <85 years while there were lower risks in older ages. These associations translated into 1.6 (95% CI -2.6, 5.9) fewer hospital-free years for chronic ACSCs between ages 60 years and 100 years. Incipient dementia was associated with increased risk of hospital admission for *acute* ACSCs (HR 1.58, 95% CI 1.42, 1.76 and 3.1 [95% CI 2.3, 4.0] fewer hospital-free years). Across specific conditions, there was a general pattern of lower risks for chronic ACSCs and higher risks for acute ones.

Our subgroup analyses suggested age-dependent associations between incipient dementia and hospital admission for ACSCs in females and age-constant associations in males (Table A2, Fig. A1 in supplement). In both sexes, incipient dementia was associated with fewer hospital-free years for any and acute ACSCs and more hospital-free years for chronic ACSCs. In subgroup with *no prior* hospital admission for ACSCs, the associations between incipient dementia and hospital admission for any and acute ACSCs were age-dependent with lower hazard in younger and oldest ages and higher hazard between these (Table A2, Fig. A2). The hazard ratio for chronic ACSCs was age-constant with a magnitude of 0.74 (95% CI 0.64, 0.86) suggesting lower risks of hospital admission for these conditions among persons with dementia compared with those without.

## 4 Discussion

In this population-based register study, we investigated the association between incipient dementia and hospital admission for ACSCs in a large cohort of persons with OA of peripheral joints. Our results revealed an age-dependent association between incipient dementia and the hazard of admission for any ACSCs with increased hazards in ages<86 years and decreased hazards in older ages. In absolute terms, incipient dementia was associated with around 6 fewer hospital-free years for any ACSCs between ages 60 and 100 years. We also found that the elevated risks were more profound for acute than chronic ACSCs.

To our knowledge, only one previous cross-sectional study investigated (as a subgroup analysis) the association between dementia and hospital admission for ACSCs in OA using 1-year of claims data from Medicare beneficiaries in the US and, consistent with our findings, reported higher rates of admissions among persons with than without dementia [18]. A few studies conducted in the general population reported higher rates of hospital admission among persons with dementia than those without [16,17,19,20]. The crude rate observed in our study (58 admissions per 1000 person-years) for people with incipient dementia is smaller than the rate reported in a study conducted among 2268 participants in Swedish National study on Aging and Care-Kungsholmen (88 admissions per 1000 person-years) [16]. This discrepancy might be due to the differences in the cohort characteristics (e.g. persons with OA vs. the general population, and younger and more males in our study), geographic location (Skåne vs. Stockholm) as well as identification of incipient dementia (register vs. a three-step procedure) which might in turn result in different levels of dementia severity. The proportion (8%) of individuals developing dementia during a mean 5 years follow up in the population-based study in Sweden [16] was similar to the proportion over about 7 years follow up in our OA cohort (8%). Increased rates and hazards of admission for ACSCs among individuals with incipient dementia might reflect the complexity induced by the disease leading to inadequacies in its management and fragmented care in ambulatory care setting [26]. This highlights the urgent need for interventions to improve the continuity of care for OA persons with dementia, including formation of interdisciplinary primary care teams and more systematic screening for cognitive impairment in primary care setting [16,27].

Higher risks of hospital admission for ACSCs among younger compared with older persons with dementia have also been reported in previous studies [21,28]. While this might reflect a better ambulatory care management among older people with dementia [21], it might also suggest a tendency toward less active interventions (treatment bias) [29] considering the fact that dementia and older age are strong independent risk factors for delirium and in-hospital complications among elderly persons [[30], [31], [32]]. In other words, even with similar or poorer quality outpatient care for older people compared with younger ones, healthcare providers’ might be reluctant to hospitalize older dementia patients due to elevated risks of in-hospital complications among them. Furthermore, the observed age-difference in the risks of ACSCs might reflect difficulties in access to healthcare services for older individuals with dementia [33]. This finding might also indicate a survivor effect, where those OA individuals with dementia who survived beyond age 86 years are likely those with mild dementia and/or with better care support and coordination [34].

Our results suggest that while incipient dementia in persons with OA was associated with an elevated risk of admission for acute ACSCs, it was generally associated with a decreased risk of admission for chronic ACSCs. Previous studies in non-OA cohorts also reported more profound associations between incipient dementia/other mental health diseases with admission for acute than chronic ACSCs [10,16,34,35]. Indeed, Saver et al. [10] identified, among a range of comorbidities, dementia as the most important predictor of hospital admission for acute ACSCs in US Medicare claims data. Consistent with our findings, a study among Medicare beneficiaries reported a lower risk of admission for COPD and heart failure in persons with Alzheimer's disease and related disorders compared with matched subjects without these disorders [19]. The elevated hazard of admission for acute ACSCs especially epileptic seizures might be attributable to shared risk factors and common underlying pathophysiological mechanisms [36]. People with chronic conditions might have more regular visits with primary care providers possibly leading to a better management of chronic ACSCs compared with acute conditions. However, this might also reflect a tendency to manage chronic conditions outside hospital (e.g. home, nursing homes) for OA patients with dementia. If the latter is the case, then our findings don't imply a better management of chronic than acute ACSCs and hence improvements in management of both these conditions are needed. In particular, given that a high proportion of people with advanced dementia reside in nursing homes in Sweden [37], improvements in quality of care provided in these institutions are urgently needed. This could be achieved through implementation of training courses, increasing nursing staff, and recruitment of specifically trained specialist nurses in nursing homes [37].

Several limitations of the current study should be acknowledged. We relied on healthcare register data, which may have misclassification and coding errors. Albeit the positive predictive value for a knee OA diagnosis is relatively high (88%) in the Skåne healthcare register [38], but there is no validation performed on dementia diagnosis in this register. In particular, persons with delayed dementia diagnosis have been included as non-exposed which might have biased our estimates. In addition, persons with mild diseases (i.e. OA and dementia) who don't seek care are not captured. Moreover, no data were available on potential confounders such as body mass index, health-risk behaviours, or health condition severity, implying the possibility of residual confounding. Small number of persons with incipient dementia in younger ages (≤70 years) resulted in HRs with wide confidence intervals for these persons in models with non-proportional hazard, which calls for caution in interpretation of the findings. There is no international standard to identify ACSCs, which limits the generalizability of the findings and cross-study comparisons. Hospital admissions for ACSCs are, in best, an (incomplete) surrogate for the quality of and access to ambulatory care. Moreover, using register data, we were unable to determine the extent to which the admissions for ACSCs were actually avoidable.

## 5 Conclusion

The results of this large population register-based study suggest that among persons with OA, incipient dementia was associated with increased risk of admission for ambulatory care sensitive conditions in ages younger than 86 years and decreased risk in older ages. In absolute terms, there were around 6 fewer hospital-free years between ages 60 and 100 years among individuals with than those without incipient dementia. These results highlight the need for improvement in access and quality of ambulatory care including continuity of care for OA patients with dementia.

## Author contributions

AK conceived and designed the study, performed the statistical analysis, and drafted the manuscript. ME participated in acquisition of data. LSL, LED and ME participated in revising the manuscript critically for important intellectual content. All authors contributed to the interpretation of the results and approved the final manuscript for submission.

## Role of the funding source

This study was supported by funds from the Greta and Johan Kock Foundation; Faculty of Medicine, 10.13039/501100004817Lund University; the Swedish Research Council; Österlund Foundation; and Governmental Funding of Clinical Research within National Health Service (ALF). The funding sources had no role in the study design, collection, analysis and interpretation of data; in the writing of the manuscript; and in the decision to submit the manuscript for publication.

## Declaration of competing interest

AK and LSL act as part-time scientific advisors for Joint Academy®, a digital self-management program for musculoskeletal diseases including osteoarthritis. LED is founder and chief medical officer at Joint Academy®. ME declares no conflict of interest.
