# Supplementary material for: Incipient dementia and avoidable hospital admission in persons with osteoarthritis
Source: Osteoarthr Cartil Open. 2023 Jan 20;5(1):100341. doi: 10.1016/j.ocarto.2023.100341 (PMC9926213; doi:10.1016/j.ocarto.2023.100341)

Table A1. List of avoidable hospitalizations (ambulatory care sensitive conditions) according to the Swedish authorities

| Diagnosis | ICD-10 codes |
|---|---|
| Chronic conditions | |
| Anaemia | D501, D508, D509 |
| Asthma | J45, J46 |
| Diabetes | E101–E108 (primary or secondary diagnosis)<br>E110–E118 (primary or secondary diagnosis)<br>E130–E138 (primary or secondary diagnosis)<br>E140–E148 (primary or secondary diagnosis) |
| Heart failure | I50, I110, J81 |
| Hypertension | I10, I119 |
| Chronic Obstructive Pulmonary Disease (COPD) | J41, J42, J43, J44, J47 (primary diagnosis)<br>J20 along with J41, J42, J43, J44, J47 as secondary diagnosis |
| Angina | I20, I240, I248, I249 |
| Acute conditions | |
| Bleeding gastric ulcer | K250, K251, K252, K254, K255, K256, K260, K261, K262, K264, K265, K266, K270, K271, K272, K274, K275, K276, K280, K281, K282, K284, K285, K286 |
| Diarrhoea | E86, K522, K528, K529 |
| Epileptic seizures | O15, G40, G41, R56 |
| Pelvic inflammatory disease | N70, N73, N74 |
| Pyelonephritis | N390, N10, N11, N12, N136 |
| Ear, nose and throat infection | H66, H67, J02, J03, J06, J312 |

Table A2. Hazard ratios and hospital free years for ambulatory-care sensitive conditions in osteoarthritis patients with incipient dementia compared with those without across subgroups.

| | Females | | Males | | No previous ACSCs | |
|---|---|---|---|---|---|---|
| | Adjusted hazard ratio (95% CI)[a] | Hospital free years (95% CI)[b] | Adjusted hazard ratio (95% CI)[a] | Hospital free years (95% CI)[b] | Adjusted hazard ratio (95% CI)[a] | Hospital free years (95% CI)[b] |
| Any ACSCs | Fig A1 | -1.2 (-4.1, 1.7) | 1.23 (1.07, 1.41) | -2.0 (-3.3, -0.6) | Fig A2 | -3.6 (-6.5, -0.7) |
| Chronic ACSCs | Fig A1 | 0.3 (-3.5, 4.1) | 0.92 (0.77, 1.10) | 0.7 (-0.7, 2.1) | 0.74 (0.64, 0.86) | 1.9 (1.0, 2.8) |
| Acute ACSCs | Fig A1 | -4.8 (-7.6, -2.0) | 1.75 (1.47, 2.08) | -4.2 (-5.7, -2.8) | Fig A2 | -5.3 (-7.7, -2.9) |

ACSCs: ambulatory care sensitive conditions.

[a]. Adjusted for sex, age, marital status, educational attainment, income, prior hospital admission for ACSCs during 1998–2009, Charlson comorbidity index during 1998–2009, and participant's as well as her/his parents' country of birth. Time-varying hazard ratios are represented in Figures A1&A2 since a single HR cannot be reported in such situations.

[b]. Adjusted for sex, age, marital status, educational attainment, income, prior hospital admission for ACSCs during 1998–2009, Charlson comorbidity index during 1998–2009, and participant's as well as her/his parents' country of birth. The difference between persons with and without dementia.

Figure A1. Age-dependent hazard ratios for the associations between incipient dementia and hospital admission for ambulatory-care sensitive conditions among women with osteoarthritis.

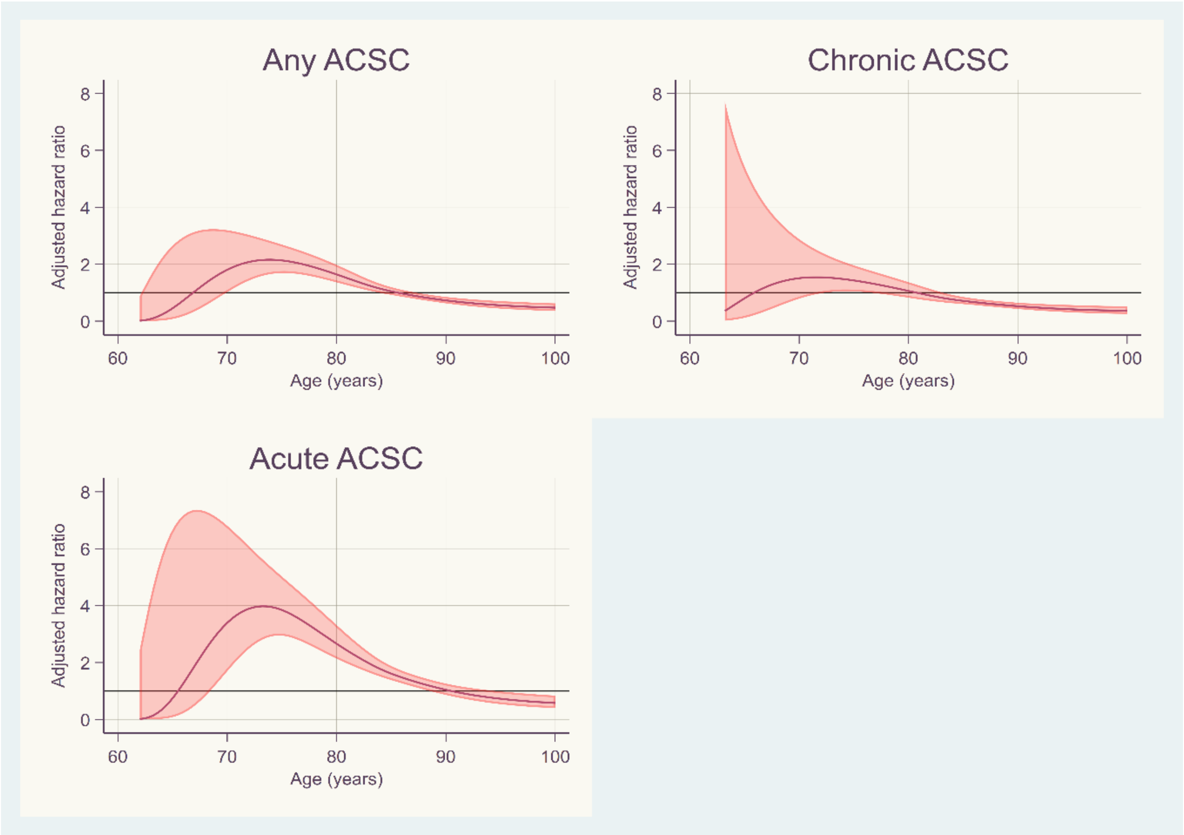

Figure A2. Age-dependent hazard ratios for the associations between incipient dementia and hospital admission for ambulatory-care sensitive conditions (ACSCs) among patients with osteoarthritis with no previous admission for ACSCs during 1998-2009.

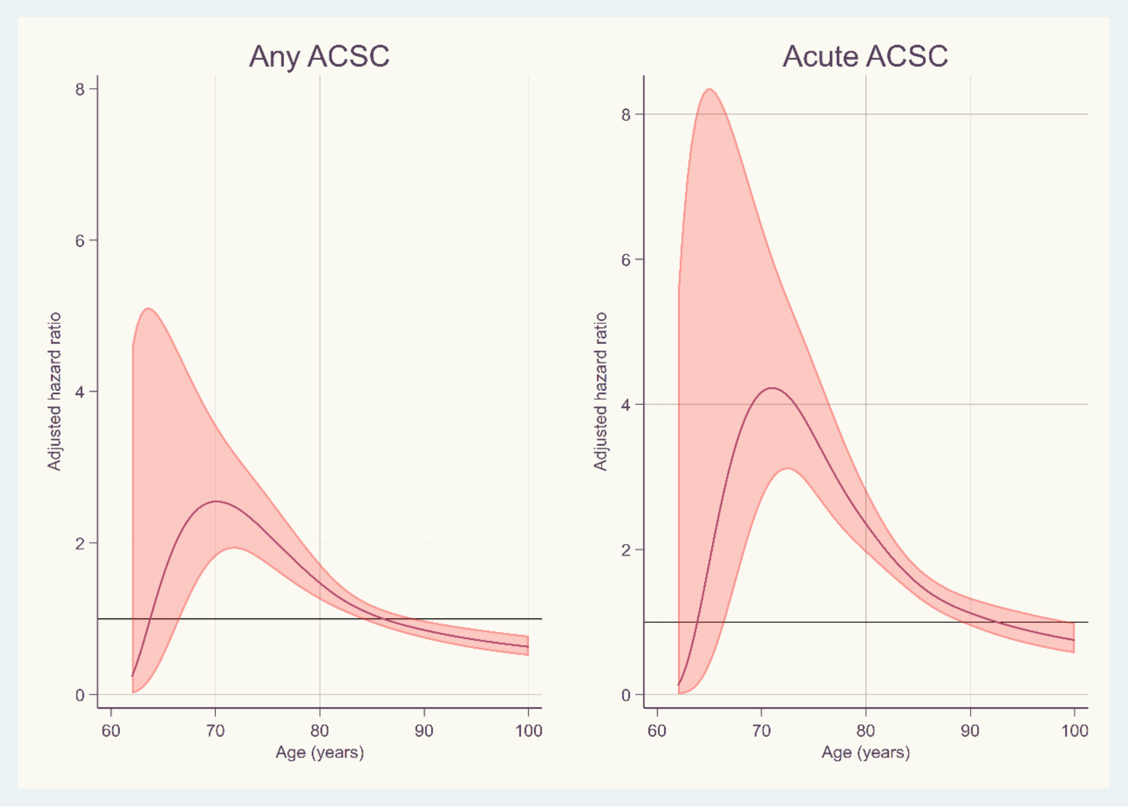

Supplement: Multimedia component 1 [file mmc1.pdf]
